# Society Meetings

**Published:** 1908-07

**Authors:** 


### SOCIETY MEETINGS.

The Lake Keuka Medical Association will hold its Ninth Annual Meeting at Grove Springs, N. Y., July 9 and 10, 1908. An elaborate program has been prepared. Among the papers in the list is one by Dr. C. C. Frederick, of Buffalo, entitled "Surgery within the Abdominal Cavity." The officers are: president, Lewis Wheeler Rose, Rochester; vice-president, Charles C. R. Jennings, Elmira; secretary and treasurer, H. B. Nichols, Pulteney. Committee of arrangements: P. L. Alden, R. G. Lawrence, Hammondsport. Registration committee: W. W. Smith, Avoca.

The Buffalo Academy of Medicine held its annual meeting Tuesday evening, June 9, 1908, at which reports were represented of the commissions on milk supply, on food supply, on inspection of schools and school children, and on smoke and noise nuisance. The following named officers were elected for the ensuing year: president, Edward A. Bowerman; secretary, Harry R. Trick and treasurer, William Irving Thornton.

The Medical Society of the County of Erie held its regular meeting Monday, June 15, 1908, in the rooms of the Society of Natural Sciences, Buffalo Library Building, with a business session at 4 P. M., devoted entirely to the regular work of the meeting; and a scientific session at 8.30 P. M. Five minute talks were made by H. C. Matzinger on Mental Changes Associated with

Prolonged Visceral Diseases; by Dewitt G. Wilcox on Tubercular Peritonitis; by S. A. Dunham on A Case of Paranoia; by Marshall Clinton on Intestinal Obstruction; by F. Park Lewis on Ophthalmia Neonatorum; by E. C. Mann on The Laity and Disease; by J. A. Gardner on A Plea for Support of the Buffalo Branch of the Society for Sanitary and Moral Prophylaxis; and by J. H. Potter on An Interesting Case.

---

THE Oregon State Medical Association held its 34th Annual Meeting, July 1, 2, and 3, 1908, at Portland. Dr. William House, formerly of Buffalo, is the secretary.

